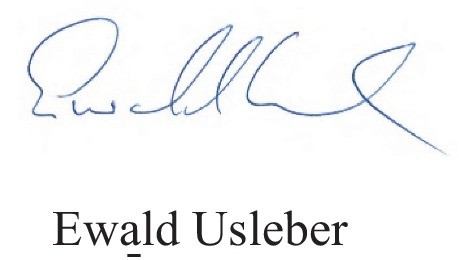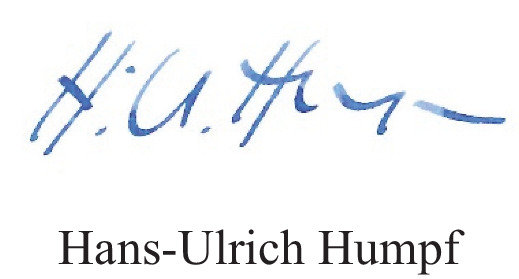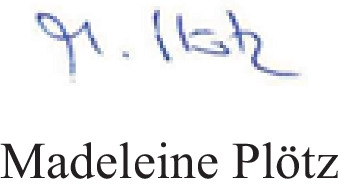# *Mycotoxin Research* in the twenty-first century: the course for the future is set

**DOI:** 10.1007/s12550-023-00475-5

**Published:** 2023-04-05

**Authors:** Ewald Usleber, Hans-Ulrich Humpf, Madeleine Plötz

**Affiliations:** 1 Dairy Sciences, Institute of Veterinary Food Science, Justus Liebig University, Ludwigstrasse 21, 35390 Giessen, Germany; 2 Institute of Food Chemistry, Westfälische Wilhelms-Universität Münster, Corrensstrasse 45, 48149 Münster, Germany; 3 Institute for Food Quality and Food Safety, University of Veterinary Medicine Hannover, Bischofsholer Damm 15, 30173 Hannover, Germany

As most of the readers of *Mycotoxin Research* may have already recognized, there was a recent change in the position of the editor in chief (EiC). Prof. Dr. Hans-Ulrich Humpf from the University of Münster has taken over this position, effective January 1, 2023, from Prof. Dr. Ewald Usleber, who served as EiC for the last 15 years. *Mycotoxin Research*, the official journal of the *Society for Mycotoxin Research* (https://www.mycotoxin.de), has continuously developed very well over the years and is now one of the leading journals in the field of research in all aspects of mycotoxins. This is reflected by a current impact factor (Clarivate Analytics, 2021) of 4.082. *Mycotoxin Research* is covered by all major scientific databases worldwide, and online access is guaranteed by our publisher, Springer/Nature. The change in the position of the EiC was already announced by the *Society for Mycotoxin Research* during the 43rd Mycotoxin Workshop 2022 in Toulouse, France (Puel et al. 2022). The retiring EiC, Ewald Usleber, would like to thank all authors, the members of the editorial board, and all reviewers, for their continuous support of our journal. Likewise, he thanks the editorial team at Springer/Nature. Without the dedication and perseverance of all these people and without the great support from the members of the society, the success story of *Mycotoxin Research* would not have been possible.

The *Society for Mycotoxin Research*, represented by the president Prof. Dr. Madeleine Plötz, on behalf of all members of the *Society*, takes the opportunity to thank Ewald Usleber for his outstanding commitment with *Mycotoxin Research* over the last 15 years. Without his continuous support, the journal would not be where it is today. The above-mentioned success of the journal is directly attributed to Ewald Usleber.

Saying this, the incoming EiC Hans-Ulrich Humpf follows in big footsteps left by Ewald Usleber. Hans-Ulrich Humpf will take up the challenge to continue the success story of *Mycotoxin Research*. Research in the field of mycotoxins is of increasing interest due to several challenges such as the consequences of climate change for agriculture, namely elevated temperatures, increased CO_2_ concentrations, and drought stress or the global shortage of grain due to the Ukraine war. The interdisciplinary scientific community is challenged to develop solutions for all these issues in order to produce enough high-quality grain and grain products without significant mycotoxin contamination to feed the world with a population of more than 8 billion people. In the light of this development, we are looking forward to publish high-quality manuscripts in *Mycotoxin Research*. As indicated on the websites of *Mycotoxin Research* (https://www.springer.com/journal/12550), the journal covers all aspects of mycotoxin research: ecology and genetics of mycotoxin formation; mode of action of mycotoxins; metabolism and toxicology; agricultural production and mycotoxins; human and animal health aspects, including exposure studies and risk assessment; food and feed safety, including occurrence, prevention, regulatory aspects, and control; environmental safety and technology-related aspects of mycotoxins; and chemistry, synthesis, and analysis of mycotoxins.

In order to cover all mentioned aspects, *Mycotoxin Research* will introduce associate editors in the near future, to cover specific topics with their expertise. Furthermore, as authors more and more demand short notification and publication time, we will significantly shorten the time from submission to first decision.

We would like to thank the authors, members of the editorial board, and the reviewers for their substantial contribution to ensure the high scientific quality and the great success of *Mycotoxin Research*. We also thank the publisher Springer/Nature with all involved coworkers for their support in all administrative questions and the fruitful and helpful collaboration.

Mark the date: we are looking forward to meeting you at the 44th Mycotoxin-Workshop, which will be held in Celle (near Hanover), Germany, from 5 to 7th of June, 2023. This year’s Workshop will be organized by the group of Madeleine Plötz from the University of Veterinary Medicine, Hanover, in cooperation with the *Society for Mycotoxin Research*. Further information will be available on the workshop website (https://www.mycotoxin-workshop.eu/).